# An ontology-driven, case-based clinical decision support model for removable partial denture design

**DOI:** 10.1038/srep27855

**Published:** 2016-06-14

**Authors:** Qingxiao Chen, Ji Wu, Shusen Li, Peijun Lyu, Yong Wang, Miao Li

**Affiliations:** 1 Center of Digital Dentistry, Peking University School and Hospital of Stomatology, 22 Zhongguancun Avenue South, Haidian District, Beijing 100081, PR China; 2 Department of Prosthodontics, Peking University School and Hospital of Stomatology, 22 Zhongguancun Avenue South, Haidian District, Beijing 100081, PR China.; 3 National Engineering Laboratory for Digital and Material Technology of Stomatology, 22 Zhongguancun Avenue South, Haidian District, Beijing 100081, PR China.; 4 Research Center of Engineering and Technology for Digital Dentistry of Ministry of Health, 22 Zhongguancun Avenue South, Haidian District, Beijing 100081, PR China.; 5 Beijing Key Laboratory of Digital Stomatology, 22 Zhongguancun Avenue South, Haidian District, Beijing 100081, PR China.; 6 Tsinghua-Rohm Electronic Engineering Hall 8-301,Tsinghua University, Beijing, 100084, PR China.

## Abstract

We present the initial work toward developing a clinical decision support model for specific design of removable partial dentures (RPDs) in dentistry. We developed an ontological paradigm to represent knowledge of a patient’s oral conditions and denture component parts. During the case-based reasoning process, a cosine similarity algorithm was applied to calculate similarity values between input patients and standard ontology cases. A group of designs from the most similar cases were output as the final results. To evaluate this model, the output designs of RPDs for 104 randomly selected patients were compared with those selected by professionals. An area under the curve of the receiver operating characteristic (AUC-ROC) was created by plotting true-positive rates against the false-positive rate at various threshold settings. The precision at position 5 of the retrieved cases was 0.67 and at the top of the curve it was 0.96, both of which are very high. The mean average of precision (MAP) was 0.61 and the normalized discounted cumulative gain (NDCG) was 0.74 both of which confirmed the efficient performance of our model. All the metrics demonstrated the efficiency of our model. This methodology merits further research development to match clinical applications for designing RPDs. This paper is organized as follows. After the introduction and description of the basis for the paper, the evaluation and results are presented in Section 2. Section 3 provides a discussion of the methodology and results. Section 4 describes the details of the ontology, similarity algorithm, and application.

Partial tooth loss is a major dental disorder that severely affects an increasing number of Chinese people and imposes significant medical, social and economic burdens^1^,^2^. Although implant treatment is regarded as the optimal approach, removable partial dentures (RPDs) remain a primary treatment for most partially edentulous patients to maintain oral function and appearance^3^,^4^. The third national dental health survey of China^1^ reported nationwide use of RPDs because RPDs cost less than other treatments, such as implants and fixed dentures.

There are several challenges associated with RPDs in China. Denture complaints have been reported, including lack of retention, pain, and functional and appearance problems^1^. Many factors may lead to discomfort in patients with RPDs^5^. Appropriate design is a priority as this greatly affects the outcome. However, it appears that poor designs are common in China and could result in discomfort and even extraction^1^. It is possible that insufficient knowledge and experience of the dentists contribute to poorly designed RPDs.

Clinical decision support systems (CDSSs) are computer systems designed to facilitate clinician decision making for individual patients^6^,^7^,^8^. There are very few such systems or models to assist in the design of RPDs. To the best of our knowledge, the only expert system in this field is the RaPiD system, which was developed in the 1990s by researchers from the UK to help dentists in the design of RPDs^6^,^9^. RaPiD, an integrated knowledge-based system that applies computer-aided design, provides guidance for appropriate design. However, the system is not intelligent enough to automatically provide a complete design of an RPD because its relevant rules cover only a small portion of the knowledge in this field. Furthermore, its rule-based approach fails to reach the necessary complexity of knowledge because of its lack of robustness and flexibility^10^.

The clinical decision support model we built is intended to help clinicians with individualized design of RPDs. The strategy of selecting components for a partial denture has been highlighted as a method to be considered for logical RPD design in dentistry^5^,^11^. Unlike CDSSs that offer diagnoses and guidelines for treatment^7^, our model aims to design specific RPDs that vary with subtle changes in patients’ oral conditions. Additionally, the design of RPDs involves the selection and location of various components composing a complete prosthesis. It is a challenge to build such a clinical decision support model. The more complex the decision to be made, the more difficult it is to build an appropriate computer model of the domain to support it^12^.

In our model, we address these challenges. The system we built in this study is called CDSSinRPD. It is designed to yield an experimental model that provides detailed and specific designs of RPDs for partially edentulous patients. It helps dentists to make clinical decisions regarding RPD design by showing the most relevant and appropriate designs for an individual patient. CDSSinRPD could provide advice and support to less experienced dentists in China.

The conceptual framework of the model is illustrated in Fig. 1. The model consists of three parts: input, process box and output.

Input includes the oral conditions of partially edentulous patients. For each patient, we must describe the oral examinations and their results in a structured way. This can be achieved using ontology, a useful tool to represent terms, concepts and the relationships among them. Our model also integrates with our electronic health record (EHR) system, which contributes patient information in that system to the input.

The process box is used to manage the input data and provide a result as the output. The process box applies an instantiation process, ontology and case-based reasoning (CBR). The instantiation process transfers the findings of oral examinations into structured terms so they can be stored in the ontology database and processed by the CBR tool. The ontology stores structured terms, the relationships between them and cases represented by the terms. The CBR is used to produce the RPD design via mathematical models using standard cases in ontology. As there is no evidence for RPD design, it is almost impossible for dentists to express their knowledge and experience as exact rules. The RPD designs and related oral examinations are based on the design criteria and philosophy taught in Peking University Hospital and School of Stomatology. Also we referred to some reference such as *An Atlas of Removable Partial Denture Design, McCracken’s Removable Partial Prosthodontics*, etc.^2^,^5^,^11^,^13^,^14^,^15^,^16^.

The output is the RPD design. It is presented in text format.

The details of the ontology and CBR are illustrated in Section 4.

The following example explains how our model works. A partially edentulous patient was missing teeth (FDI notation) 35, 36, 37, and 38. Teeth 33, 34, 44, 45, and 46 were healthy with no caries or periodontal issues. The residual ridge of each missing tooth was normal and stable. No soft tissue existed above the ridge. According to the findings on the articulator, there was adequate inter-ridge space for a prosthesis. There was also adequate inter-occlusal space for rest seats and rests on 33, 34, 44, 45, and 46. Information collected in clinical examination was entered into our system. The ontology in the process box managed the information in a structured form. The CBR identified five cases that were similar to this one, and their corresponding RPD designs were output. The design of one RPD is summarized in Table 1.

The output of our model is presented in text format. The model would be a very useful reference for dentists in drawing RPD design sketches in routine practice.

To build such a model, we first needed a tool that (1) represents domain knowledge in a structured and formalized way, (2) integrates with the EHR used in Peking University Hospital and School of Stomatology for data extraction and (3) stores standard cases in the form of instantiation for further calculation of similarity. We used the ontology as a vital means for knowledge representation. The ontology can also be viewed as a dataset to store standard cases in the form of instantiation^17^. Details of the ontology are provided in Section 4. Unlike rule-based reasoning, CBR involves feature extractions and mathematical approaches that can significantly determine the robustness and flexibility of the model. A cosine similarity algorithm is applied to identify the most relevant cases from the dataset in our ontology and to output the designs of extracted cases.

## Evaluation and Results

The reliability and accuracy of our clinical decision support model are contingent on several factors, including ontology construction, case base, natural language processing (NLP) and the similarity algorithm. Thus, the entire model was evaluated to assess its performance. Methods of evaluation in information retrieval were applied^18^,^19^.

A total of 104 patients diagnosed as being partially edentulous were selected at random from our EHR data warehouse for evaluation. After the CDSSinRPD was applied, a set of RPD designs was compiled for each patient, with their similarity values calculated by a cosine similarity algorithm in descending order. Only designs with values above the threshold or at a top rank were considered similar to the final design. In this study, we chose the top 5 cases as the final results. Professionals in Peking University Hospital and School of Stomatology selected 5 standard cases of each test sample as the correct results, and these were used for the evaluation.

Precision is one of the most frequently used and basic metrics for evaluating model effectiveness^19^. Precision is relevant when comparing retrieved cases. It shows the fraction of retrieved cases that are similar to the final design. For example, in our study the system retrieved 5 cases, 3 of which were correct results. In this example, the precision was 3/5 = 0.6. An area under the curve of the receiver operating characteristic (AUC-ROC) is created by plotting true-positive rates against the false-positive rate at various threshold settings. It integrates an ROC curve with the highest value of 1 and the lowest value of 0. AUC-ROC represents comprehensive conditions of a binary classifier system. A higher value indicates better system performance. A mean average of precision (MAP) is capable of measuring the orders in which the retrieved cases are presented. Values are on the interval of 0 to 1. MAP values are higher when relevant cases are returned at the top of the returned cases. A normalized discounted cumulative gain (NDCG) is a measure of ranking quality. Relevant cases are ranked according to their relevance. It is based on the assumption that highly relevant cases are much more useful when appearing at the top of retrieved cases. Therefore, highly relevant retrieved cases at the top would receive a high NDCG value, with a maximum of 1.

**Figure 2:**
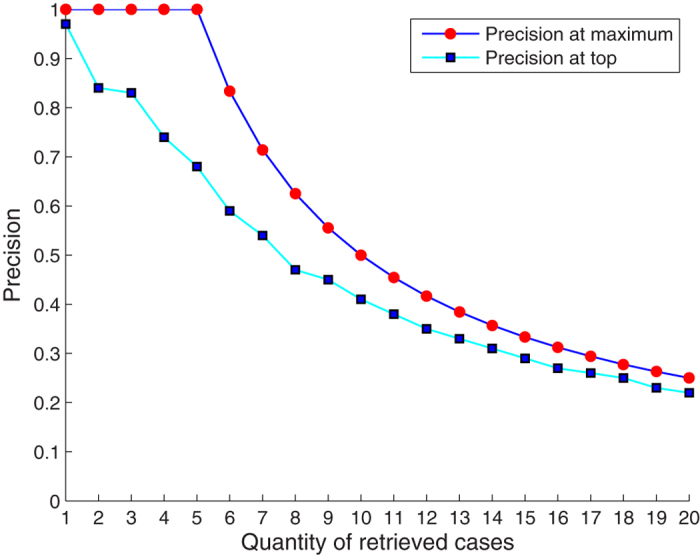
Precision of the top 1 to 86. The quantity of the retrieved cases means the number of cases retrieved by our model. Precision is the metric measuring the percentage of relevant cases in the retrieved cases. Precision at the top shows precision values at a different number of retrieved cases, from one to twenty in our model. Precision at the maximum shows the ideal precision values at each number of retrieved cases.

**Figure 3:**
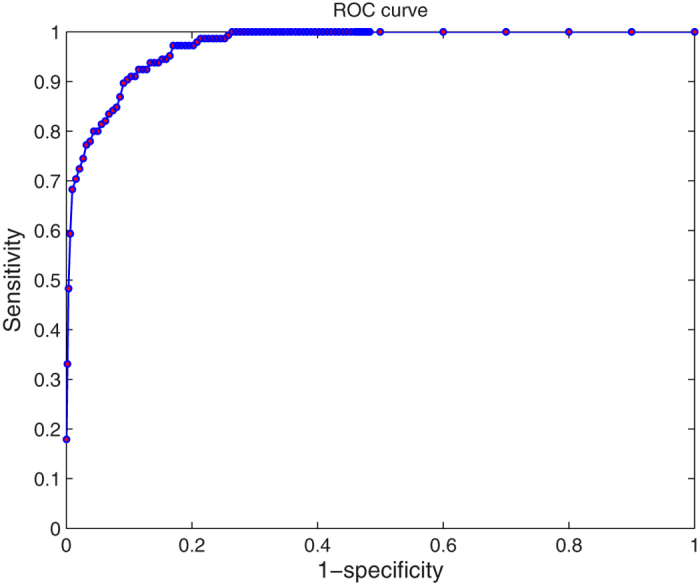
Receiver operating curve (ROC). An ROC curve plots the true-positive rate or sensitivity against the false-positive rate or (1–specificity). Sensitivity is another term for the true-positive rate. (1–specificity) means the false-positive rate.

Precision at the top computes the fraction of the relevant cases at the top of the retrieved cases. The precision at 5 was 0.67. The precision at the top 1 to 86 is shown in Fig. 2. The MAP value was 0.61, showing a good performance in retrieving the relevant cases at the top. To evaluate the accuracy of our model, an AUC-ROC was calculated based on our results. Figure 3 shows that the overall area under the ROC was 0.96, which is considered very successful. The NDCG represents the performance of returning highly relevant cases at the top. The value was 0.74.

## Discussion

RPDs are a major treatment for partially edentulous patients. The design of RPDs is of great importance and demands judicious decisions. With the aid of CDSSs, the design procedure could be much easier and achieve more accuracy. The model described in this paper represents initial research and merits further development. The evaluation results show the promise of our methodology’s performance and suggest that our model is feasible and reliable.

In our trial, we exploited ontology as a tool for knowledge construction. In the area of decision support, ontology often plays a role in knowledge representation. In combination with rules, knowledge representation is a source of domain knowledge and supports reasoning in applications^17^. However, in our research, the complex and ambiguous nature of knowledge prevents simple reasoning directed by the ontology to make a complete design. The ontology in our model is a knowledge representation tool compatible with the EHR system. The ontology we constructed also acts as a database storing standard cases in the form of instantiations.

CBR is another major element of our work. This problem-solving method uses existing knowledge and experience^20^. Cosine similarity is normally used in information retrieval dealing with textual information. However, in our work it is first applied to calculate the similarity values between different features. The algorithm often calculates the frequency of extracted terms to find the most similar cases in an information retrieval of EHR data^21^. In our trial, the cosine algorithm calculated the property values of the classes in our ontology. In our model, the properties all derive from the classes of the ontology, and the experts set the weights accordingly.

Based on the metrics of the evaluations and results mentioned above, we can see that our model is capable of offering reasonable treatment designs for users. The metrics also indicate that our algorithm is quite accurate and adaptable. Because feature extraction is based on ontology, the good results also show a successful ontology construction. The precision shown in Fig. 2 indicates that the model could find the most relevant cases. Compared with the maximum precision under different quantities of retrieved cases, the precision at the top in our model is close to the optimal line. The MAP of 0.61 reflects the ranking of the most relevant cases near the top of retrieved cases. The AUC-ROC is a general evaluation for the entire model regarding the ontology construction, EHR data extraction, case base and similarity algorithm. In our trial, the AUC-ROC of 96% reveals that the model is acceptable, with quite high accuracy. The NDCG is strongly influenced by the graded relevance and positions of relevant cases. The NDCG of 0.74 demonstrates our model’s ability in returning highly relevant cases at the top positions.

The evaluation results show that our method is efficient in RPD design. Because the domain knowledge in dentistry has the same logic and representation format, our methods can also be applied to other fields in dentistry. For example, the ontology could be used in prosthodontics with suitable modification. CBR has wide application, and its success in our model suggests the possibility of applications in other areas.

This is the first type of work in this field, and there are still some aspects to be improved. During the CBR process, more properties can be considered to improve the accuracy of the final results. This will require further development of the methodology. Based on the ontology we built, new methods might be offered. For example, optimization methods might be used to select reasonable designs. The idea of optimization is to score each patient’s oral condition step by step and then choose reasonable design components. The designs would then be ranked by machine learning.

## Methods

### Ontology construction

**Figure 4:**
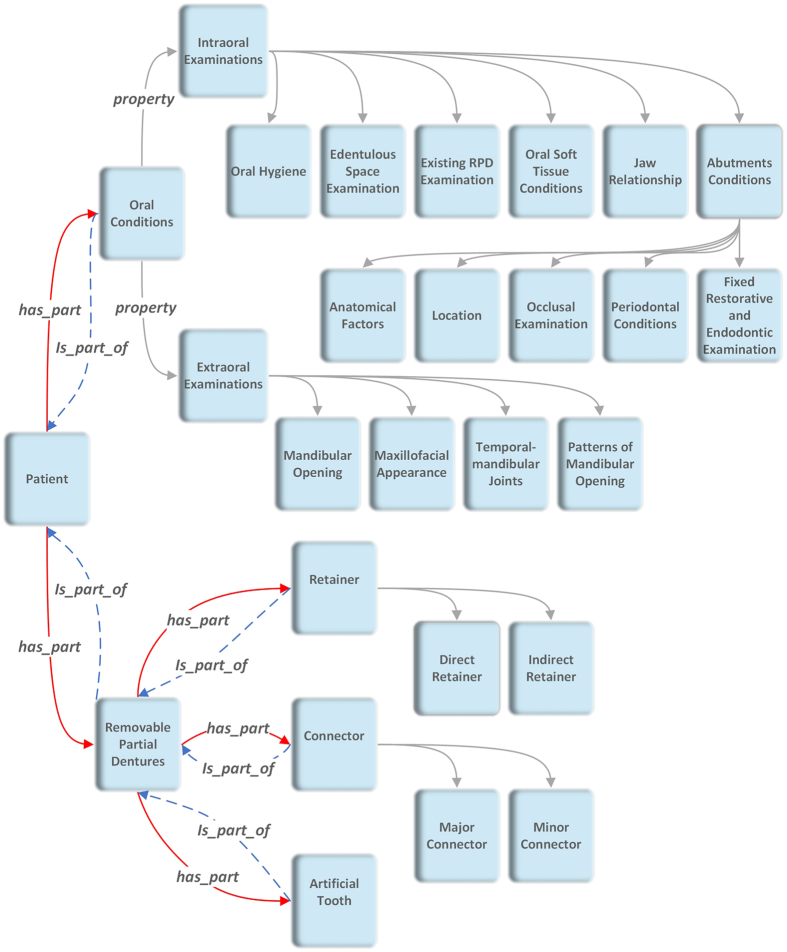
Framework of the top-level classes of ontology. This figure shows the main classes of our ontology. *Patient* is the top level. *Oral Conditions* represents related oral examinations, and *Removable Partial Dentures* represents the components of a complete prostheses. Both of them are in the subclass of *Patient*.

The input of our model is information about patients’ oral conditions obtained in numerous oral examinations. There are logical relationships among such examinations, which are illustrated in Fig. 4. For instance, there are two major categories: extraoral examinations and intraoral examinations. Five examinations marked as subclasses constitute the intraoral examination. Those five examinations are at the same level in the subclasses. The intraoral examination is the class above those subclasses. Specific examinations are properties of those subclasses and at the bottom of the hierarchy. For example, degree of mobility is one property of the subclass *Periodontal Conditions*. It is the same when we represent the RPD designs. The output is the RPD designs, which means the choice of category of each component (e.g., ring clasp, Aker clasp) and the location of each component (e.g., the occlusal rest is put on the mesial occlusal surface of the mandibular right first molar). As an example of a class representing the categories and locations of those components, *Removable Partial Dentures* contains three subclasses: retainer, connector and artificial tooth. Direct retainer and indirect retainer are subclasses of retainer. Specific components, such as continuous clasp, are marked as a subclass at the bottom level.

Our model, *CDSSinRPD*, has an implemented ontology to construct knowledge structure. Our knowledge structure consists of two parts: the input knowledge structure and output knowledge structure, which we explained above. The ontology is a useful tool that plays an important role in biomedical research because of its function in knowledge management, data integration and decision support^17^,^22^,^23^,^24^. Protégé is a software used to create ontologies^25^,^26^. In Protégé, we use the web ontology language (OWL) to implement our ontology.

In order to use a common language and well-defined structured terms in ontology, we refer to glossaries in our field: “The Glossary of Prosthodontic Terms” (8th edition) and *Mosby’s Dental Dictionary* (*2th edition*). A book entitled *McCracken’s Removable Partial Prosthodontics* is also used to ensure that our terms are correct, understandable and tractable^13^,^15^,^16^. We selected nearly 100 concepts from those references as unambiguous descriptions of oral conditions. The concepts reflect the findings of general examinations and some more detailed examinations. Those detailed examinations, which encompass such features as undercut position and tooth alignment, are vital to the design but are rarely recorded on paper during clinical treatment. The top-level classes of our knowledge graph are depicted in Fig. 4.

The ontology has two classes: *Oral Conditions* and *Removable Partial Dentures. Oral Conditions* include the results of patients’ oral examinations, which are the foundation of clinical decisions, including diagnosis and treatment planning. Thus, the design of RPDs is based on oral examinations in ontology. The class *Removable Partial Dentures* contains subclasses referring to components of RPDs. Their properties show the location and other information regarding those parts. Therefore, the design of an RPD would be represented through *Removable Partial Dentures* and the internal properties. To connect the input for one patient with its output, we construct a class, *Patient,* which consists of *Oral Conditions* and *Removable Partial Dentures. Patient* has object properties *is_part_of* and *has_ part. is_part_of* is a containing relation of two classes. For example, “*oral conditions” “is_part_of” “patient”* means the former term is included in the latter one. *has_ part* is the inverse relation of *is_part_of*.

The *Oral Conditions* class refers to the results of oral examinations of patients included in our model. The properties of this class and its subclasses are described by data properties, which represent all specific examinations, and their values are treated as examination results.

The *Removable Partial Dentures* class describes the categories and locations of the RPD components. Categories of components are built as subclasses of *Removable Partial Dentures.* The locations of each component are built as properties of each subclass.

In addition to the object property *is_part_of*, other properties are constructed to represent other relations; for example, *before* represents the location of the abutment and *belong_to* and *has* its inverse. To represent an individualized design of an RPD, data properties in *Removable Partial Dentures* are built to show details of components such as position of the retentive portion of the arms, types of teeth loss and so on.

Functioning as a case base, the proposed ontology also stores all standard cases in a form of instantiation for potential reasoning. The reason is that such a case base is formalized and can be easily matched to data extracted from an EHR. We referred to *An Atlas of Removable Partial Denture Design*, which covers more than 95% of the situations and ensures that we cover most of the possible situations^14^. In our model, 164 standard cases of Kennedy I and Kennedy II class of mandibular disorders are stored in the case base. Each standard case is matched with an RPD design corresponding to its oral conditions and is used in our reasoning process. Our ontology is also compatible with EHR data. Those data are extracted, using NLP, from the data warehouse of the EHR system in the Prosthodontics Department of Peking University Hospital and School of Stomatology . All the extracted information is normalized and organized by our ontology for further calculation in our reasoning process.

### Knowledge base: CBR and related algorithms

A traditional knowledge-based CDSS would be rule-based^7^. However, rule-based methods cannot completely satisfy our needs for the following reasons. First, it is almost impossible for experts to express their knowledge and experience as exact rules because RPD design is a complicated process. In addition, the domain knowledge is not clear enough and rather complex. Second, rule-based methods require many rules. Too many rules would slow down the system and also tend to contradict one another. Third, the methods are not robust. Because the system is limited to rules, a new problem cannot be solved if none of the rules apply. Moreover, oral examinations are not always Boolean formats. They could be rating scales or numerical values. Such knowledge could not be represented by rules.

In our research, we use CBR^18^,^20^. By comparing input information with cases existing in ontology, CBR identifies the most similar cases in the system and outputs the treatment of those cases as results. Although oral conditions vary from one patient to another, there is a degree of similarity. By comparing the oral conditions in different cases, we can select designs suitable for the aspects of those cases that are similar.

The cosine similarity algorithm is used to calculate the similarity between new patients and standard cases in the ontology. The value of the similarity is defined by the cosine value of the angle between two vectors. Let *A* = (*A*_1_, …, *A*_*n*_) and *B* = (*B*_1_, …, *B*_*n*_) be two n-dimensional vectors. Equation 1 defines the cosine similarity.


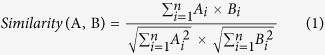


The value of similarity (A, B) measures the degree of similarity between the two vectors. It is widely used in high-dimensional positive spaces^21^. In our trial, for each property of oral conditions in ontology, we define a vector. The *i*th component of the vector is defined by the value of the property corresponding to the *i*th tooth. Each tooth corresponds to one component of the vector. For example, in the ontology the value of the property of whether or not a tooth is missing is set to 0 or 1, respectively. Because there are normally 16 teeth in one arch, the dimension of the vector is 16 (i.e., n = 16).

The position of missing teeth is a significant factor in designing RPDs. Experts believe that the farther back a tooth is located in an arch, the more important it is for the design of RPDs. Therefore, we divide the 16 teeth in each arch into four groups: anterior teeth (incisors excluded), incisors, premolars and molars. Each group has a different role in the design of RPDs. Based on the principles above, 16 components of the vector can be divided into 4 groups. Each group can be regarded as a new vector. Each new vector has a different weight. In our method, we first calculate the cosine similarity of each group separately. The similarity value is calculated between the vectors of a standard case in the ontology and vectors of an input new patient. Then, a score is obtained by aggregating these values using a weighted linear combination. The score is used to measure the degree of similarity between the two cases. Its value leads to an estimate of how suitable the previous design of RPDs is for the current patient. The higher the score, the greater the similarity between the two cases.

The cosine similarity in this paper does not take into account the frequency of each term^18^,^21^.

Based on the relevant scores, we ranked the RPD designs in descending order of similarity. We chose the top five designs as our ultimate designs. The system retrieves a list of ranked designs of RPDs as the final decision. The output of our model is in the form of text with illustrations of their classification and locations.

### Ethics Approval

The study was approved by the Bioethics Committee of Stomatological Hospital of Peking University, Beijing, China. (no. PKUSSIRB-201520019). All experimental protocols were approved by the Bioethics Committee of Stomatological Hospital of Peking University, Beijing, China. All experiments were performed in accordance with approved guidelines and regulations. Informed consent was obtained from all subjects.

## Additional Information

**How to cite this article**: Chen, Q. *et al.* An ontology-driven, case-based clinical decision support model for removable partial denture design. *Sci. Rep.* **6**, 27855; doi: 10.1038/srep27855 (2016).

## Figures and Tables

**Figure 1:**
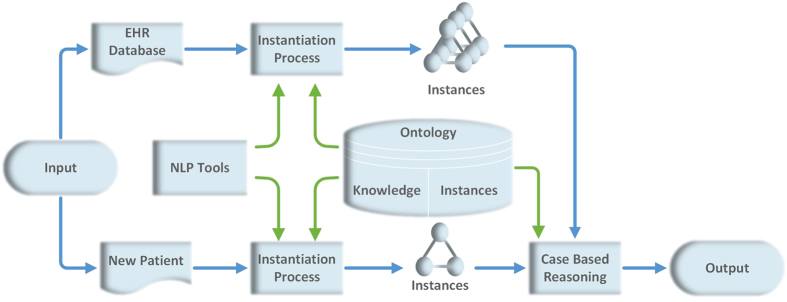
Overview of the CDSSinRPD model. The framework of our model. The EHR database and new patient are both sources of input. After the instantiation process, which was guided by the ontology that we constructed, the input data were standardized in a form of instance. Case-based reasoning began through cosine similarity between instances in the ontology and input. The treatment of similar cases informed the final output.

**Table 1:** A schematic view of the CDSSinRPD output.

Design 1 for Patient A:
**Abutments:**
Teeth 33, 34, 43, 46
**Direct retainer:**
**(a)** RPI clasp location: tooth 34 rest: on the mesial occlusal surface
**(b)** Aker clasp location: tooth 46 rest: on the mesial occlusal surface
Note: the tips of two arms are towards mesial direction
**Indirect retainer:**
**(a)** Lingual rest location: cingulum on tooth 33
Note: it shares the same minor connector with RPI clasp on tooth 34
**(b)** Occlusal rest location: mesial occlusal surface of tooth 43
**Major connector:**
**(a)** Lingual bar location: extend from 34 to 45
**Artificial tooth:**
(**a**) Semi-anatomic teeth location: teeth 45, 46, 47 number: 3
